# Attitudes Toward Four Levels of Self-Driving Technology Among Elderly Drivers

**DOI:** 10.3389/fpsyg.2021.682973

**Published:** 2021-06-23

**Authors:** Timo Lajunen, Mark J. M. Sullman

**Affiliations:** ^1^Department of Psychology, Norwegian University of Science and Technology, Trondheim, Norway; ^2^Department of Social Sciences, University of Nicosia, Nicosia, Cyprus

**Keywords:** autonomous vehicle, attitudes, preferences, elderly drivers, SAE levels, sustainable transportation

## Abstract

Automatization and autonomous vehicles can drastically improve elderly drivers' safety and mobility, with lower costs to the driver and the environment. While autonomous vehicle technology is developing rapidly, much less attention and resources have been devoted to understanding the acceptance, attitudes, and preferences of vehicle automatization among driver groups, such as the elderly. In this study, 236 elderly drivers (≥65 years) evaluated four vehicles representing SAE levels 2–5 in terms of safety, trustworthiness, enjoyment, reliability, comfort, ease of use, and attractiveness, as well as reporting preferences for vehicles employing each of the four levels of automation. The results of a repeated-measures ANOVA showed that the elderly drivers rated the SAE level 2 vehicle highest and the fully automated vehicle (SAE 5) lowest across all attributes. The preference for the vehicle declined as a function of increasing automatization. The seven attributes formed an internally coherent “attitude to automatization” scale, a strong correlate of vehicle preference. Age or annual mileage were not related to attitudes or preferences for automated vehicles. The current study shows that elderly drivers' attitudes toward automatization should be studied further, and these results should be taken into account when developing automated vehicles. The full potential of automatization may not be realized if elderly drivers are ignored.

## Introduction

It has been estimated that 1.35 million people are killed, and 50 million are injured each year in road accidents around the world (WHO, 2018). Previous research has suggested that driver error plays a role in around 90% of road traffic crashes (Sabey and Taylor, 1980; International Organisation for Road Accident Prevention, 2011; Smith, 2013). Furthermore, research from the U.S. has estimated that the average commuter is delayed by 38 h a year due to traffic congestion and that traffic congestion generates an additional 25.4 billion kilograms (56 billion pounds) of CO_2_ emissions each year (Schrank et al., 2012).

The replacement of the current vehicle fleet with autonomous vehicles has the potential to both improve safety and reduce vehicle emissions associated with congestion. The substantial number of autonomous vehicles will allow us to make transportation systems safer, efficient, and less pollutant (Cartenì, 2020). Researchers have predicted that the large-scale use of automated vehicles will significantly reduce the number of fatalities and injuries caused by road traffic crashes (Fagnant and Kockelman, 2015; Blanco et al., 2016; Simoes, 2018). Furthermore, the wide-scale use of autonomous vehicles may also improve pedestrians and cyclists' safety, resulting in increased use of active modes of transport (Millard-Ball, 2018). Autonomous vehicles can be expected to improve safety, especially among drivers with impaired driving abilities. Old drivers are overrepresented in intersection crashes (Clarke et al., 2010), demonstrating that deterioration of vision, cognitive impairment and particular age-related illnesses are reflected on driving abilities (Langford et al., 2005). For example, Wolfe et al. (2020) showed that 55–69 or old drivers needed a significantly longer time to detect and correctly respond to a road hazard. Compared to young drivers, elderly drivers have a safer driving style characterized by slower speed, more gradual acceleration and deceleration, fewer violations and longer headways (Horberry et al., 2004; Langford et al., 2005). Since older drivers' crashes are related to impaired driving ability, we can expect vehicle automatization to be particularly helpful for elderly drivers and lead to a significant decrease in drivers' crash risk.

In addition to safety, vehicle automatization and fully autonomous vehicles have a great potential for making transport system more efficient, energy-efficient and, thus, environmentally sustainable. Automatization provides a higher degree of optimization of mobility solutions and leads to greater flexibility, takes advantage of the “sharing concept,” and provides solutions that have lower greenhouse gas emissions (Cruz and Sarmento, 2020). Vehicle automatization can lead to the transformation of transportation from individually owned vehicles to the development of Mobility-as-a-Service (MaaS) approach in which the aim is efficient, environmentally sustainable mobility for all citizens, including such groups as the elderly who might have reduced mobility due to impaired driving ability. A recent interview study conducted in the Greater Manchester region (U.K.) by Zandieh and Acheampong (2021) showed that older adults perceive that automated vehicles can enhance their physical activity, promote social interaction and offer stress-free door-to-door mobility, which underlines the role of automated vehicles in MaaS for providing higher mobility for the elderly. Finally, it is also expected that there will be significant reductions in congestion due to improvements in traffic flow and several other potential changes in the pattern of vehicle use (Fagnant and Kockelman, 2014; Greenblatt and Saxena, 2015; Bajpai, 2016).

There are a number of different levels of automation, and also a number of definitions of self-driving technology have been proposed. The most well-known definitions have been proposed by the National Highway Traffic Safety Administration (NHTSA, 2020) and SAE (SAE J3016, 2014). The NHTSA (NHTSA, 2020) proposes five different levels of automation, which are:

Level 0 The human driver does all the driving.
Level 1 An advanced driver assistance system (ADAS) on the vehicle can sometimes assist the human driver with either steering or braking/accelerating, but not both simultaneously.
Level 2 An advanced driver assistance system (ADAS) on the vehicle can actually control both steering and braking/accelerating simultaneously under some circumstances. The human driver must continue to pay full attention (“monitor the driving environment”) at all times and perform the rest of the driving task.
Level 3 An automated driving system (ADS) on the vehicle can itself perform all aspects of the driving task under some circumstances. In those circumstances, the human driver must be ready to take back control at any time when the ADS requests the human driver to do so. In all other circumstances, the human driver performs the driving task.
Level 4 An automated driving system (ADS) on the vehicle can itself perform all driving tasks and monitor the driving environment—essentially, do all the driving—in certain circumstances. The human need not pay attention in those circumstances.
Level 5 An automated driving system (ADS) on the vehicle can do all the driving in all circumstances. The human occupants are just passengers and need never be involved in driving.

There is currently a small but growing body of research that has examined public attitudes toward self-driving technology. This research has mainly focused on a range of predictor variables, including demographic variables, specific psychological characteristics (e.g., sensation-seeking and risk-taking), interest in self-driving technology, desire for self-driving technology and willingness to pay for this technology (Becker and Axhausen, 2017).

Previous research has investigated the relationship between demographic variables and interest in using self-driving technology. These studies have found that males are significantly more interested in using self-driving technology (e.g., Payre et al., 2014; Schoettle and Sivak, 2014; Bansal et al., 2016; Hohenberger et al., 2016; Zmud and Sener, 2017) and are willing to pay more for self-driving technology than females (Kyriakidis et al., 2015; Bansal et al., 2016). Previous research has also found that younger drivers were more interested in using self-driving technology and that younger drivers were prepared to pay more for this technology than older drivers (Kyriakidis et al., 2015; Bansal et al., 2016; Hohenberger et al., 2016). A recent survey conducted in eight European countries showed that the elderly are concerned mostly about the safety of automated driverless vehicles and would prefer to travel in autonomous vehicles with the presence of a human supervisor (Kyriakidis et al., 2020). Similar findings showing that older and disabled persons are more concerned about reliability and safety of the autonomous vehicles, than younger and non-disabled persons, have been found in several earlier studies (Nielsen and Haustein, 2018; Gabrhel et al., 2019; Huff et al., 2019; Liu et al., 2019; Robertson et al., 2019; Liang et al., 2020). The cost of automatization also seems to concern older people. In addition to concerns for safety, elderly citizens are less willing to pay for automatization or worried about the cost (Kyriakidis et al., 2020; Zandieh and Acheampong, 2021). In a telephone survey study among 501 older (≥65 years) adults, 78.2% of the respondents stated that they would not pay any additional amount for the driverless features (Oxley et al., 2019).

However, the findings for age are not as consistent as for sex, with several studies finding no relationship (e.g., Payre et al., 2014; Zmud and Sener, 2017). It should also be noted that a substantial percentage of the population would not be prepared to pay anything extra for self-driving vehicles (Schoettle and Sivak, 2014; Kyriakidis et al., 2015; Daziano et al., 2017).

Classen et al. (2019) reviewed 28 studies about the effect of in-vehicle information systems (IVIS) and ADAS on older drivers' convenience, comfort or safety. These findings indicated that the IVIS or ADAS enhanced safety and mitigated age-related performance decrements, as long as the system were simple enough to use and thus did not compromise one's cognitive workload. While the review did not directly assess the impact of automated driving systems beyond ADAS or self-driving technology, we can expect the benefits of SAE levels 4 and 5 technology to be even more drastic. Elderly drivers form a group, which could benefit considerably from automated driving in terms of safety and increased mobility. As Knoefel et al. (2019) point out, in their theoretical article about the possible benefits of semi-autonomous vehicles among the elderly, the loss of a driver's license can have drastic adverse effects on elderly drivers' general health and mental and social well-being. The decline in mobility due to driving cessation may have a direct negative impact on social connectedness with friends and relatives (Mezuk and Rebok, 2008), volunteering and employment (Curl et al., 2013), as well as independence and life management (Adler and Rottunda, 2006; Al-Hassani and Alotaibi, 2014). Driving cessation among the elderly seems to contribute to a variety of health problems, especially depression (Chihuri et al., 2016), cognitive decline (Yamin et al., 2015), and higher risks of admission to long-term care facilities and mortality (Chihuri et al., 2016). These findings imply that automated driving systems and self-driving technology have great potential not only to improve safety among elderly citizens but also to extend the years of physically and mentally healthy life.

Previous research on the acceptance of automated driving systems among elderly drivers have focused mainly on the acceptance of individual ADAS features (e.g., Adaptive Cruise Control, Forward Collision Warning) or on the acceptance of self-driving vehicles in general. In the ADAS evaluation studies, drivers evaluated the attractiveness of each individual ADAS feature, which does not provide a clear indication about the level of automation older drivers prefer. For example, a driver might like to have an automatic emergency braking system installed, while he or she might not want to have adaptive cruise control. The self-driving vehicle studies have used survey designs in which respondents were asked to indicate their attitude to “self-driving vehicles” in general (see Rahman et al., 2019). While these kinds of studies might provide a useful insight into the attitudes and acceptance of the highest SAE level vehicles, they do not tell us which level of automatization is most preferred by elderly drivers.

Therefore, the present research aimed to investigate the attitudes and preferences of elderly drivers toward automatization in SAE levels 2–5. In this way, we asked drivers to compare the different SAE levels and, thus, indicate which SAE level they found the most appealing for their future car. This type of comparative study, between the different SAE levels of automated driving, has not previously been conducted and thus, there is an incomplete understanding of elderly drivers' preferences for automated vehicles.

## Methods

### Participants

Participants were recruited in Australia, Canada, the USA, and the U.K. by advertising the study on Facebook and Instagram. The advertisement was titled “Automation in cars–good or bad?” and the respondents were motivated with the question “What do you think about automation in vehicles?” to click a link to the web survey (SurveyMonkey) page. The advertisement was seen (at least once) by 18,564 people, and the link clicked by 548 people. The data were collected between June 15 2020, and July 3, 2020.

In order to take part, participants had to be at least 18 years old and have a valid license to drive. Participants did not receive any monetary or other rewards for taking part. The sample consisted of 350 respondents. From 548 people clicking the advertisement, 350 responded; hence, the respondence rate was 64%. From this sample, a sub-sample of ≥65-year-olds was separated. We chose not to limit the age of participants to the elderly because that could have caused a pre-selection bias, the age being the most important criterion instead of being a driver. The younger participants were not included in the study because the sample size was too small and because the study was aimed at investigating elderly drivers.

In total, 236 elderly individuals took part (38 women, 197 men), and they were mostly from the U.K. (54.7%), followed by the USA (26.3%), Canada (5.1%), and Australia (13.6%). Hence, the sample was predominantly British and male, which should be taken into account when evaluating the results. The mean was 72.6 years (SD = 5.4 years; range 65–94 years). They had held a license to drive for an average of 53.2 years and reported an average annual mileage of 15,167 km (SD = 33,086 km).

### Materials

The questionnaire was hosted using Survey Monkey and consisted of two sections. The first section asked questions about the participant's demographic and descriptive variables (i.e., age, sex, license status, license tenure, annual mileage, and whether they had access to a car). The second section of the questionnaire consisted of questions in relation to autonomous vehicles. In this section, four different autonomous vehicles were described, which correspond to four of the five types of autonomous vehicles described by (SAE J3016, 2014) and correspond to Level 2 (Occasional Self-Driving), Level 3 (Limited Self-Driving), Level 4 (Full Self-Driving under limited conditions), and Level 5 (Full Self-Driving under all conditions).

For each of the four car types, participants were presented with a short description of the self-driving capabilities of the vehicle and examples of what the car was able to do. For example, SAE Level 3 vehicle was described in the following way:

Car B. Conditionally automated car: “eyes off the road in certain conditions”

- The car can take care of everything while driving in limited conditions (e.g., slow-moving traffic).
- You can get engaged in other activities like watching a film or using your phone, but not sleeping.
- You do not have to keep your hands on the steering wheel all the time, but have to be able to take full control of the car if the system fails or if the conditions are not suitable for automation.
- You have the option to manually override the system.
- The car has normal controls like a steering wheel, accelerator, and brake pedal.

Since we can expect high variability among the respondents' familiarity with ADAS and ADS (Oxley et al., 2019), the descriptions were designed to be as comprehensive and simple as possible with practical examples.

Following this description, participants were then asked to report the degree to which seven descriptors (safe, trustworthy, enjoyable, reliable, comfortable, easy to use, attractive) reflected driving the vehicle above. Responses were made on a five-point Likert scale (1 = None at all to 5 = A great deal). Participants were also asked to report on a five-point scale (1 = Not at all interested to 5 = Extremely interested) how interested they would be in having each of the four types of self-driving technology described.

### Procedure

The survey was advertised on Facebook in the UK, Canada, the USA, and Australia. Interested participants clicked on the advert and were taken to the Survey Monkey page. The first page informed participants what the study was about, what they were being asked to do, and obtained informed consent.

## Results

### Evaluation of the Vehicles Representing SAE Levels 2–5

**Figure 1:**
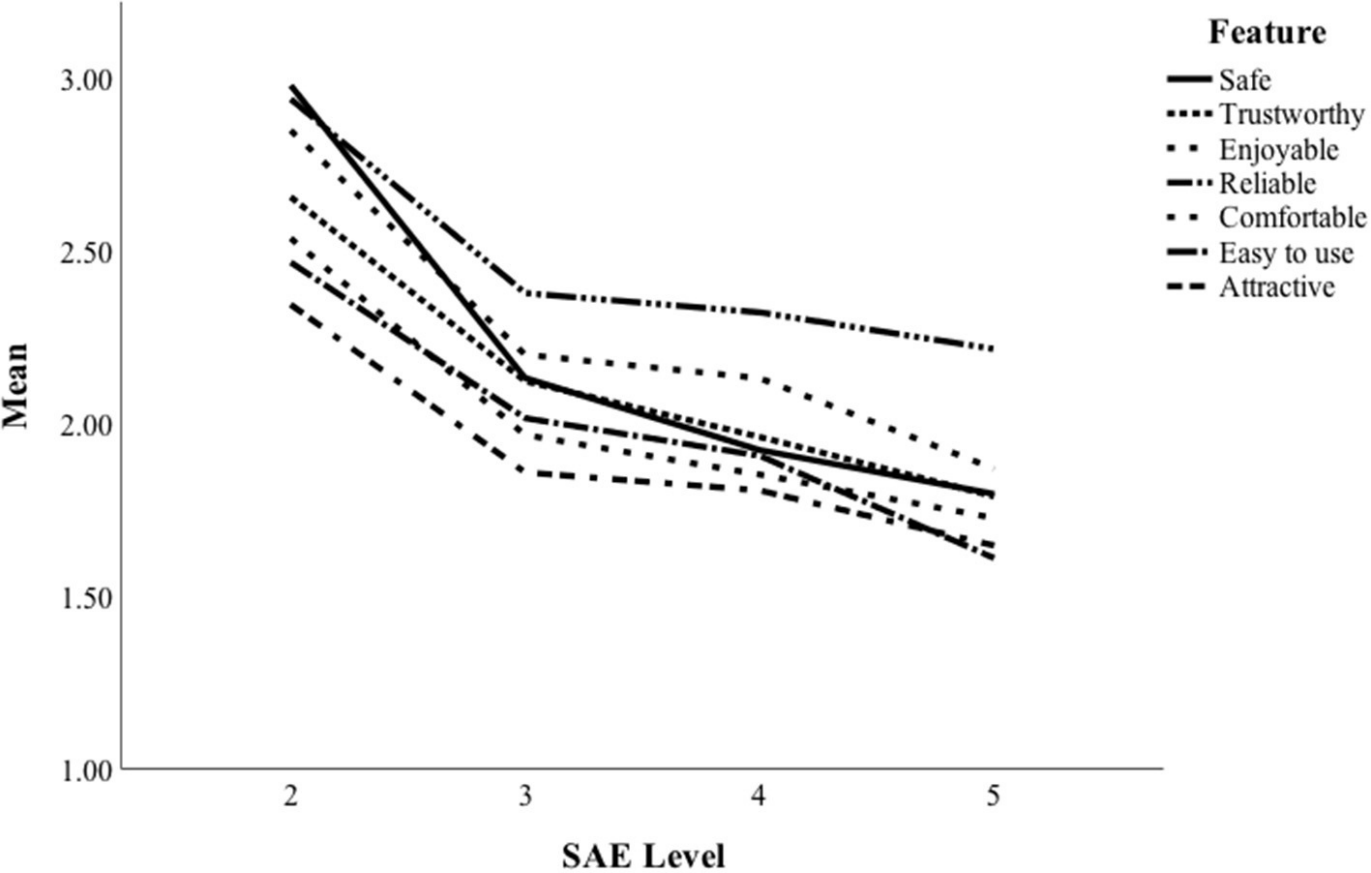
Attitudes to vehicles (SAE levels 2–5).

The participants' evaluations of the four vehicles, representing SAE levels 2–5, in terms of seven features, can be seen in Figure 1. A repeated-measures ANOVA showed that the main effect of SAE level was statistically significant for the evaluations of safety [*F*_(3, 167)_ = 40.63, *p* < 0.001, η^2^ = 0.42], trustworthiness [*F*_(3, 163)_ = 19.97, *p* < 0.001, η^2^ = 0.27], enjoyment [*F*_(3, 166)_ = 18.96, *p* < 0.001, η^2^ = 0.26], reliability [*F*_(3, 165)_ = 21.72, *p* < 0.001, η^2^ = 0.28], comfort [*F*_(3, 163)_ = 25.07, *p* < 0.001, η^2^ = 0.32], ease of use [*F*_(3, 163)_ = 14.62, *p* < 0.001, η^2^ = 0.21], and attractiveness [*F*_(3, 162)_ =18.71, *p* < 0.001, η^2^ = 0.26]. Figure 1 shows a decline in all seven features measured with an increase in automation so that the most favorable vehicle type belongs to SAE 2, while the fully automated vehicle was the least favored type. The Bonferroni adjusted pairwise comparisons showed that the vehicle representing the lowest SAE level (2) scored higher than the vehicles from the higher SAE levels in each feature assessed. The SAE level 2 car scored significantly (*p* < 0.001) higher than the SAE level 3 car in terms of safety, trustworthiness, enjoyment, reliability, comfort, ease of use, and attractiveness. The pairwise comparisons also indicate that the SAE levels 3 and 4 were evaluated equally enjoyable (*p* = 0.411), reliable (*p* = 0.233), comfortable (*p* = 1.000), easy (*p* = 1.000), and attractive (*p* = 1.000). Moreover, the SAE levels of 4 and 5 were evaluated equally safe (*p* = 0.338), trustworthy (*p* = 0.180), and easy to use (*p* = 1.000). These comparisons may indicate that after some level of automatization, the increase in automatization does not make a difference for the users. Nevertheless, the comparisons show that the lowest level of automatization (SAE level 2) was the most favorable in all features measured.

### Combined Attitude Score in SAE Levels

Figure 1 shows that different features evaluated for each SAE level followed the same pattern in terms of the degree of automatization. One reason for this finding might be that the respondents could not make a significant conceptual difference between the seven adjectives used for evaluation. To investigate the relationships between the seven features within the SAE classes, Pearson product-moment correlations were calculated. The correlations ranged between 0.67 and 0.81 for SAE 2; 0.70–0.92 for SAE 3; 0.66–0.95 for SAE 4; and 0.65–0.93 for SAE 5. Correlations of this strength might indicate that the features actually form one “attitude to automatization” scale instead of being separate indicators.

The seven features were subjected to four principal component analyses (separately for each SAE level). Both the Scree plot and “eigenvalue greater than one” criteria yielded a clear one-component solution for each SAE level. The single-component named “attitude to automatization” explained 75.9, 81.4, 81.8, 83.2% of the variance within SAE 2, SAE 3, SAE 4, SAE 5, respectively. The reliability analyses (Cronbach's alpha) confirmed the results of the principal component analyses: the “attitude to automatization” sum scale showed high internal consistency for SAE 2 (α = 0.95), SAE 3 (α = 0.96), SAE 4 (α = 0.96), and SAE 5 (α = 0.97). It seems that the higher the level of automatization, the more variance the one-component solution explained, and the more internally consistent the summed scale was.

**Figure 2:**
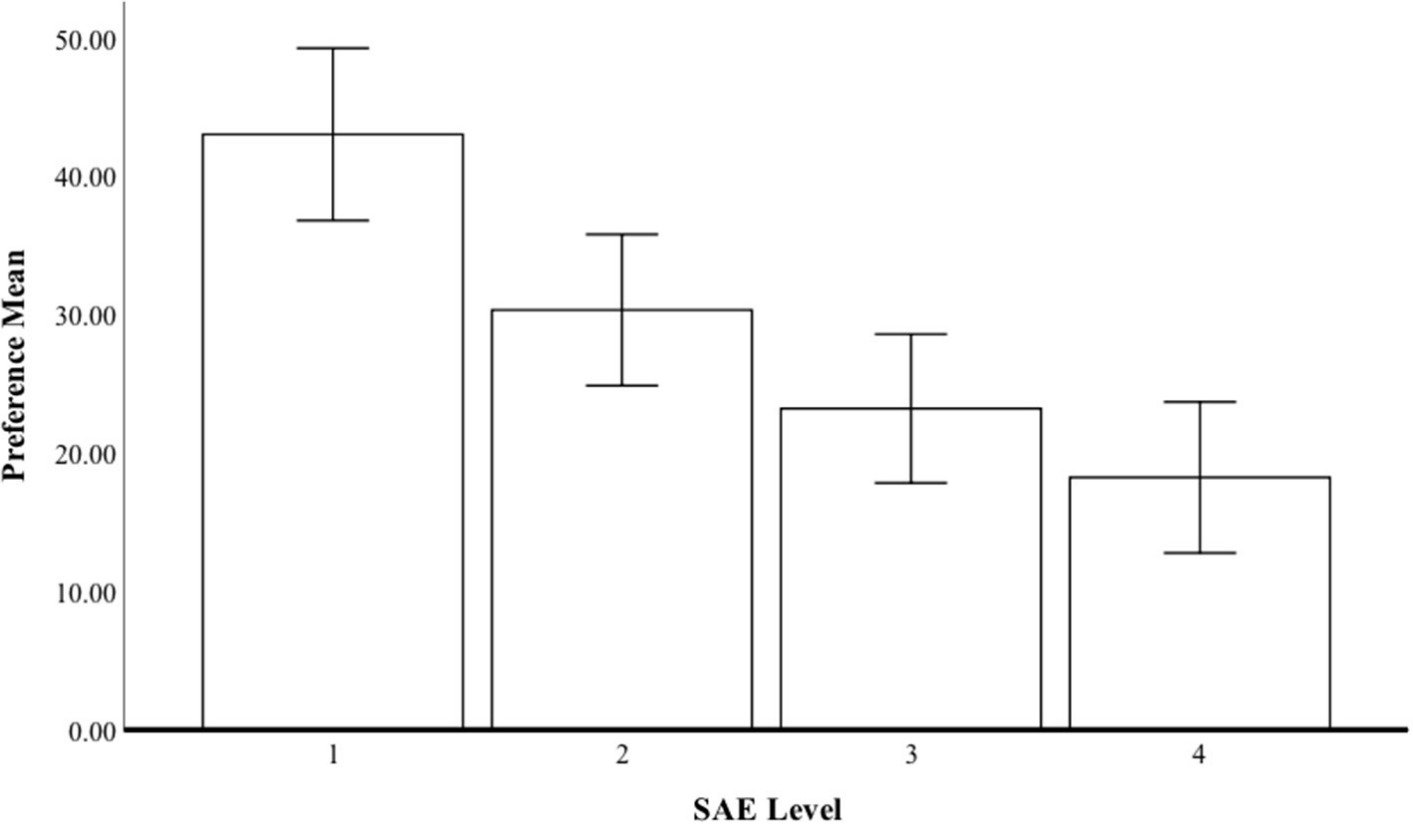
Attitudes to automatization (SAE levels 2–5).

Figure 2 shows the attitude mean score for each SAE level and the confidence interval (95% level). A repeated-measures ANOVA showed that the main effect of the SAE level was statistically significant for the evaluations of safety {Wilks' Lambda = 0.638; [*F*_(3, 168)_ =31.73, *p* < 0.001, η^2^ = 0.36]}. Attitude to SAE 2 was more positive than to the other SAE levels, while attitudes to SAE level 3 were statistically significantly higher than to level 5 (*p* < 0.001). The respondents seemed to clearly prefer as low a level of automatization as possible.

### Preferred Level of Automatization

**Figure 3:**
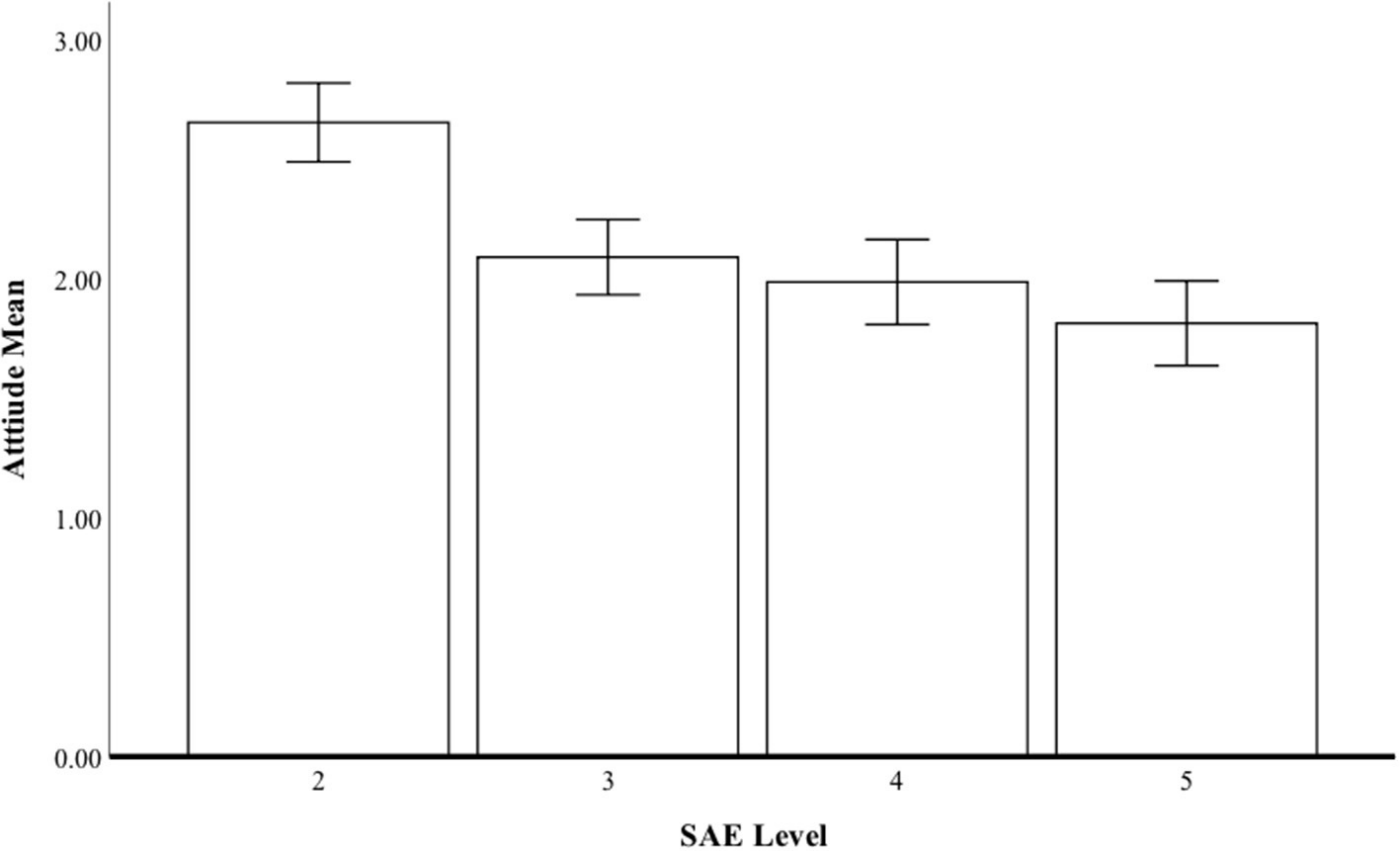
Preference for the vehicles (SAE levels 2–5).

The respondents were asked to indicate (from 0 to 100%) the degree to which they prefer to drive four cars, each representing a different SAE level. Figure 3 shows that the degree of preference decreased according to the level of automatization {Wilks' Lambda = 0.684; [*F*_(3, 128)_ = 19.72, *p* < 0.001, *h*^2^ = 0.32]. Attitude to SAE 2 was more positive than to the other SAE levels. Attitudes to SAE level 3 were significantly higher than to level 5 (*p* < 0.001). The respondents seemed to clearly prefer the lowest level of automatization possible.

### Correlations Between Background Factors, Attitudes, and Preferences in Different SAE Levels

Table 1 presents the Pearson product-moment correlations between age, annual mileage, attitudes, and the preference scores for SAE levels 2–5. Table 1 shows that the age of the respondent and annual mileage driven did not correlate significantly with either the attitudes or preference scores for each SAE level. The positive attitudes to different SAE levels had relatively high intercorrelations: the closer the levels were to each other, the stronger the correlation was. This could be explained simply with a minimal difference between two neighboring levels. If a respondent prefers the amount of automatization provided by level 2, he or she is more likely to accept level 3 more than level 4, etc. Interestingly, the highest correlation (0.87) was between a positive attitude to level 4 and 5. In terms of positive attitudes, “Limited Self-Driving” (Level 4) and “Full Self-Driving under limited conditions” (Level 5) seemed to be very similar to each other.

**Table 1:** Correlations between background variables, attitudes, and SAE preferences.

	**1**	**2**	**3**	**4**	**5**	**6**	**7**	**8**	**9**
1. Age	1.0								
2. Mileage	−0.11	1.0							
3. Attitude to SAE 2	0.02	0.02	1.0						
4. Attitude to SAE 3	0.02	−0.02	0.69^**^	1.0					
5. Attitude to SAE 4	−0.06	−0.06	0.51^**^	0.85^**^	1.0				
6. Attitude to SAE 5	−0.11	−0.09	0.39^**^	0.70^**^	0.87^**^	1.0			
7. Preference of SAE 2	−0.11	0.11	0.63^**^	0.44^**^	0.37^**^	0.23^*^	1.0		
8. Preference of SAE 3	−0.06	0.10	0.52^**^	0.68^**^	0.63^**^	0.44^**^	0.79^**^	1.0	
9. Preference of SAE 4	−0.05	−0.05	0.44^**^	0.68^**^	0.77^**^	0.66^**^	0.56^**^	0.81^**^	1.0
10. Preference of SAE 5	−0.03	−0.12	0.38^**^	0.62^**^	0.77^**^	0.86^**^	0.33^**^	0.47^**^	0.73^**^

* *p < 0.01*;

** *p < 0.001*.

Positive attitudes were strong predictors of a preference for the SAE level concerned. The relationship between attitudes and preference seemed to increase as a function of automatization, being lowest in SAE 2 level (*r* = 0.63) and strongest in SAE level 5 (*r* = 0.86). Hence, preferring a more highly automatized vehicle reflects stronger positive attitudes than the preference for a vehicle with a lower level of automatization.

## Discussion

Automatization is one of the fastest-growing fields in transportation, with a rapid increase in the resources invested. Automatization can be expected to have profound effects on the way we travel, the environment, and our society (Hohenberger et al., 2016). Since 94% of crashes can be attributed to human error (Singh, 2015), and increased automation level can be expected to result in a huge leap forward in traffic safety. A fully autonomous (SAE level 5) vehicle virtually removes the driver from the driver's seat and thus eliminates the possibility of human error. This would, in particular, reduce injuries and fatalities among those drivers with impaired driving ability, such as the elderly or those with special needs. Together with an increase in the number of electric vehicles, automatization is also likely to reduce the impact of transportation on the environment, while at the same time reducing the cost for the individual (Burns et al., 2013). Automated and optimized transportation systems applying the latest driverless technology are very likely to reduce unnecessary trips and, thus, the costs for the individual road user (Burns et al., 2013). Automatization in general, and especially fully automated cars, might also contribute to the mobility of older citizens and people with disabilities to enhance their individual mobility and, consequently, their participation in society (Fagnant and Kockelman, 2015). Vehicle automatization is an essential part of the Mobility-as-a-Service approach, in which the aim is to find the most optimal solution for an individual's travel needs, taking into account cost-effectiveness and environmental sustainability (see Cruz and Sarmento, 2020). In sum, automatization and automated vehicles have great potential to make transportation safer, more inclusive, and more sustainable.

So far, most of the discussion about automated vehicles has focused on vehicle technology and less on the adoption of the technology among different driver groups (Madigan et al., 2016). In their qualitative review, Becker and Axhausen (2017) reviewed studies in which factors related to the acceptance of SAE Levels 4 and 5 automated vehicles were investigated. Becker and Axhausen (2017) concluded that the general opinion or intention to use automated vehicles varied considerably among the studies. In a study conducted in the U.K., only 18% of the 1,001 respondents regarded the development as “important” (Ipsos MORI, 2014). Other studies about automated vehicles have reported relatively low acceptance rates for automated vehicles (Becker and Axhausen, 2017). One crucial factor affecting attitudes to automated vehicles is a respondent's age: older drivers have more critical attitudes to the automatization of vehicles than younger respondents do. Automated vehicles seem to be the most popular among young people and those in urban environments (Hohenberger et al., 2016; Becker and Axhausen, 2017).

One explanation for the negative attitudes to automatization among elderly drivers might be that most of the studies—all studies included in the review by Becker and Axhausen (2017)—asked opinions about highly automatized vehicles (SAE levels 4 and 5) without including the lower SAE levels. In this way, most of the studies cannot describe the change in attitudes as a function of automatization. In our study, we asked elderly drivers (≥65 years) to evaluate vehicles from SAE levels 2–5, which allowed us to investigate the change in attitude and preference in terms of SAE level. The results showed that the elderly drivers clearly preferred SAE level 2, compared to the more advanced technology. This was found both in the seven attitude ratings as well as when the preference for the vehicle was asked. SAE level 2 can be considered as “old fashion driving with ADAS,” in which the human driver monitors the driving environment. SAE level 2 is in full contrast to SAE levels 3–5, in which the vehicle monitors the environment and makes decisions without human interference. Interestingly, the decrease in attitudes and preferences occurred between levels 2 and 3. Moreover, a relatively steady decrease was found in all features, including safety, trustworthiness, enjoyment, reliability, comfort, ease of use, and attractiveness. It should be noted that the price of the car was set to be equal (i.e., at the same price), and thus the respondents could have chosen a fully automated car for the same price as the level 2 vehicle. Therefore, it seems that the elderly drivers in this study simply dislike automatization beyond SAE level 2.

A common way of measuring attitudes toward automated vehicles is to describe a highly automated vehicle (SAE 4 or 5) and then ask the respondents to indicate the degree to which they find such a vehicle safe, reliable, etc., how much the respondent is ready to pay for such technology or has an intention to use it (Becker and Axhausen, 2017). The assumption is that the features listed are somewhat independent of each other, i.e., the drivers can differentiate between the characteristics. The results of the present study show that the seven attitudes had high intercorrelations and, in fact, formed a single factor scale with a clear unifactorial structure and high internal consistency. Moreover, all seven attitude items seemed to function similarly in terms of SAE levels (degree of automatization). This finding questions the common practice of treating attitude variables as separate factors and might indicate that the drivers evaluate automated vehicles holistically and are not able to assess different aspects of this new technology. This is understandable since most drivers cannot have had the first-hand experience of vehicles belonging to SAE levels 3–5. In our dataset of elderly drivers, it seems that the seven attitudes reflected a general attitude to SAE levels instead of measuring each attitude separately.

In the present study, the respondents preferred to drive SAE 2 level vehicles. Interestingly, neither age (within the age group) nor annual driving experience were related to their vehicle preferences. The only strong predictor of preference for the vehicle was the attitude score; the higher the SAE level was, the stronger predictor that attitude was.

Some limitations should be acknowledged when evaluating the results of the present study. Firstly, only SAE levels 2–5 were included in the study. This strategy was chosen to avoid a lengthy survey, which could have compromised reliability. Since the SAE level 2 can still be considered a level in which the driver is fully in control, including levels 0 and 1 appeared redundant. It would have been interesting, however, to see if the decline in attitudes and preferences started at level 1 or if level 2 was the most preferred level since the advanced driver assistance systems (ADAS) make driving easier while also allowing the driver to have full control. Including at least SAE 1 (SAE 0 can be seen as an exception these days, and only some classic cars belong to this level) would have served as a natural reference point. In future studies following the same design, all SAE levels 1–5 should be included. Moreover, the survey did not include questions about the vehicle the respondents were currently using and, therefore, we could not investigate how the actual user experience of ADAS could influence the acceptance of the higher SAE levels. A survey study by Rahman et al. (2019) indicated that older adults familiar with self-driving vehicles were more likely to have a favorable perception of them. In a study by Crump et al. (2016), the driver perceptions of safety when driving vehicles with ADAS weakened following repeated exposure, while with more extensive exposure to ADAS, a heightened appreciation of the ADAS was reported by an older driver, i.e., those who would benefit from assistance the most. While ADAS is conceptually different from the automated vehicle, these results may indicate that extensive exposure to automated driving can make it more acceptable among the elderly. It should be noted, however, that familiarity can be related to interest in technology and cars and does not require owning a vehicle with many ADAS features. In order to minimize the effect of familiarity with ADS, the descriptions of the different SAE Levels were made as comprehensive as possible and included examples. Nevertheless, in future studies, the respondent's exposure to vehicle automatization should be controlled at least by asking about the features of their current vehicle combined with questions about their satisfaction with the current car. In addition to direct experience, knowledge about automatization could be measured in future studies.

Secondly, our study included only elderly drivers. A comparison sample of young or middle-aged drivers would have provided an informative control, and thus a larger study that includes all age groups should be conducted. Earlier studies show that automated vehicles are preferred by typically male, young, highly educated, and those who live in large urban areas (Nielsen and Haustein, 2018; Gabrhel et al., 2019; Huff et al., 2019; Wang and Zhao, 2019). The resistance to automated vehicles may be partly explained by the lower risk preference among the elderly: older drivers expect a much higher safety level from the new technology than younger drivers do before accepting it (Liu et al., 2019; Wang and Zhao, 2019). In future studies, it would be interesting to compare young and middle-aged drivers to older drivers in the acceptance of different SAE levels.

Thirdly, the study sample was collected in June and July 2020 using Facebook and Instagram ads and an internet-based survey, which guarantees the highest level of anonymity but does not allow the calculation of a proper response rate and a detailed description of the sample. While the usual respondent characteristics, such as age, sex, license tenure, access to a vehicle, annual mileage, and country of residence were recorded, we did not record the respondents' socio-economic status, income or travel patterns (exposure to urban, rural, and motorway driving) because of the need to keep the survey short. It should be remembered that the vehicle is chosen according to purpose and, therefore, in future studies, the travel patterns should be asked. An elderly driver using his or her car only for short shopping trips might not see this new technology as necessary as being as someone with high mileage in various conditions. Since it can be expected that income is related to willingness to pay for ADS (Kyriakidis et al., 2020; Zandieh and Acheampong, 2021), in this study, the cost of ADAS and ADS features were defined as “cost-free” in order to eliminate the effect of socio-economic background. While this constraint might be theoretically sound, it is still possible that the cost of new technology plays a role in preferences. We chose not to ask about income level because that kind of sensitive information can reduce drastically the willingness to participate in the study. Interestingly, the respondents preferred the lowest SAE level, despite the fact that they could have chosen a much more technologically developed vehicle for exactly the same price. This means that there is something inherently unappealing in the high levels of automatization, irrespective of the cost. Future studies should investigate whether psychological factors, such as fear of novelty, concerns about mastering new technology, feeling a lack of control or enjoyment related to active driving, have a role in their preference for less advanced technology.

Lastly, this study was based on an internet sample. While this kind of sample can reach people who do not usually participate in studies, there are some clear limitations. Answering on-line surveys is limited only to those who use the internet and social media. Elderly drivers who use social media might also be more interested in technology and more capable of using it. Moreover, the sample in this study is predominantly male. While both males and females use social media, it seems that men are more interested in vehicle technology than women. Other sampling methods with a higher share of females could have resulted in different results.

## Conclusions

The present research highlights the importance of studying the acceptance and adoption of automated vehicles in different driver groups. Elderly drivers would be one of the groups to benefit the most from automated vehicles, because driving or riding in an SAE level 4 or 5 level vehicle would undoubtedly be safer and less stressful than driving in a traditional vehicle without the help of automatization. In addition to safety, level 5 automated vehicles could prolong the time that older people remain mobile. This study, however, shows that the elderly drivers' attitudes and the preference declines steadily as a function of increasing automatization. If this issue is not addressed, this finding may compromise the future benefits of automatization among the elderly.

Automated vehicle technology is one of the few application areas in which safety, environmental sustainability and high service level (high mobility) can be achieved. It seems, however, that the potential of new technology may be compromised because of the low acceptance rate among elderly drivers. One way to increase acceptance is to first apply autonomous driving in public transport, e.g., buses and the metro so that the users get familiarized with the technology without the need to actively deciding about it (Gabrhel et al., 2019). While young drivers are ready to accept new technologies, older drivers need more time to adjust.

While vehicle automatization has taken vast leaps forward in recent years, it is clear that the one-eyed focus on technology has ignored one of the most crucial facts: technology can benefit potential users only if the users decide to adopt the technology in the first place. If only techno-enthusiasts, or transportation companies reducing personnel costs, purchased self-driving vehicles, the true benefits of ADS will be marginal, despite the substantial investment in the development of this technology. ADAS, ADS and self-driving vehicles can particularly benefit those who have compromised cognitive capacities, such as many elderly citizens. Since ADS can significantly increase the years of healthy living and life quality for the elderly, manufacturers developing ADS and self-driving vehicles should take into account drivers with special needs, such as the elderly. In the present study, the elderly drivers preferred SAE Level 2, despite the many benefits of the higher levels. This finding indicates that elderly drivers should be taken more into account when designing vehicles employing ADS and when informing the public about the benefits of self-driving vehicles.

## Data Availability Statement

The raw data supporting the conclusions of this article will be made available by the authors, without undue reservation.

## Ethics Statement

Ethical review and approval was not required for the study on human participants in accordance with the local legislation and institutional requirements. Written informed consent for participation was not required for this study in accordance with the national legislation and the institutional requirements.

## Author Contributions

All authors have contributed equally in every stage of the study, including planning, data collection, analyses, and preparation of the manuscript.

## Conflict of Interest

The authors declare that the research was conducted in the absence of any commercial or financial relationships that could be construed as a potential conflict of interest.
